# Stain control with two experimental dentin hypersensitivity toothpastes containing spherical silica: a randomised, early-phase development study

**DOI:** 10.1038/s41405-019-0016-x

**Published:** 2019-06-06

**Authors:** Stephen Mason, Sarah Young, Mako Araga, Andrew Butler, Robert Lucas, Jeffery L. Milleman, Kimberly R. Milleman

**Affiliations:** 1 GSK Consumer Healthcare, St George’s Avenue, Weybridge, Surrey KT13 0DE UK; 2 GSK Consumer Healthcare, 184 Liberty Corner Road, Warren, NJ 07059 USA; 3 Salus Research, 1220 Medical Park Drive, Fort Wayne, IN 46825 USA

**Keywords:** Oral hygiene, Tooth brushing, Gingival recession, Dental epidemiology

## Abstract

**Aims:**

To determine in human participants whether toothpastes containing small quantities of a novel spherical silica, added to provide enhanced cleaning properties, could achieve similar or greater extrinsic dental stain removal compared to toothpastes containing standard dental abrasive silica concentrations.

**Materials and methods:**

One hundred and twenty-three adults with extrinsic dental stain were randomised to one of four parallel groups for 8 weeks’ twice-daily brushing with an experimental toothpaste containing either 0.5% or 1% spherical silica (with relative dentin abrasivity [RDA] of ~38 and ~58, respectively), or marketed toothpastes containing either 6% (RDA ~ 36) or 16% (RDA ~ 166) standard abrasive silica. The objective was to evaluate the ranking order in extrinsic dental stain removal at Week 8, as measured by MacPherson modification of Lobene stain index Area × Intensity.

**Results:**

Small treatment differences were observed between toothpaste formulations. The ranking order in extrinsic dental stain removal was: experimental 1% spherical silica toothpaste >16% standard abrasive silica toothpaste >6% standard abrasive silica toothpaste >experimental 0.5% spherical silica toothpaste. Toothpastes were generally well tolerated.

**Conclusion:**

This early-phase development study suggests that toothpaste formulations with low concentrations of a novel spherical silica abrasive with high-cleaning capability are generally well tolerated and appropriate for further development.

## Introduction

Stain-causing substances from, for example, dietary sources or tobacco, can bind to proteinaceous compounds in plaque or pellicle and lead to the appearance of tooth discolouration.^1–4^ Toothbrushing can remove dental plaque if carried out properly,^5^ but many people brush using an inadequate technique and for less than the 2 min recommended to achieve adequate tooth surface cleaning.^6^ Toothpastes formulated with chemical or physically abrasive agents can aid plaque biofilm disruption and reduce stain formation.^5,7–11^ However, the ability of a toothpaste to control extrinsic dental stain needs to be balanced with its potential to deleteriously affect the tooth surface, particularly for individuals with exposed dentin and subsequent dentin hypersensitivity.^12^ This is especially important as tooth wear is increasing across an ageing adult population.^13^

Physical abrasives often used in toothpastes include insoluble phosphates, carbonates, alumina and silicas. The ability of an abrasive to affect stain removal is influenced by particle shape, hardness, concentration, distribution and size.^12,14^ Such abrasives are relatively hard, water insoluble, inert compounds effective at mechanical cleaning that impart a relative dentin abrasivity (RDA) to the toothpaste formulation. Toothpaste formulations range from RDA 30 to 250, with an upper acceptable limit of 250;^15^ however, high-cleaning (abrasive) formulations often have an RDA >150 that may be abrasive to dentin with prolonged usage, something of concern to those with dentin hypersensitivity.^7,12,16^

The cleaning function of a toothpaste formulation can be increased by chemical cleaning including compounds such as sodium tripolyphosphate (STP).^5^ Such agents facilitate stain removal and control stain build-up by acting as chelators that bind strongly to the tooth surface, reducing the adhesive force of absorbed proteins^4^ and desorbing salivary proteins from enamel.^17,18^ This increases a toothpaste’s stain-removing efficacy without increasing abrasivity and can target dentition areas less accessible to physical abrasives or toothbrush bristles.^19–22^

With the goal of maintaining cleaning ability while lowering abrasiveness (to develop a toothpaste suitable for people with dentine hypersensitivity), a novel toothpaste formulation was recently developed and its clinical stain removal performance was evaluated. This toothpaste contained no abrasive silica (AS) and 5% STP as a cleaning and stain removal agent (RDA ~ 10).^20,21^ This current work examines a different scientific direction to achieve a similar outcome and utilises novel micronised abrasive spherical silica (SS) that can achieve similar RDA levels than conventional AS but at notably lower concentrations. Toothpaste formulations vary immensely, but AS is typically included in concentrations around 20% weight for weight (w/w) and can be utilised up to 55% w/w.^5,23,24^

It is hypothesised that the narrow size distribution of SS particulates offers a greater number of cleaning particles, comprising the appropriate physical characteristics of particle size, shape and hardness, in contrast to standard AS at the same concentration, thus facilitating removal of debris and residual stain while conferring less abrasivity. In vitro studies have shown that SS used at extremely low concentrations from 0.1% to 5% w/w may achieve a similar degree of stain and plaque removal to that demonstrated with higher concentrations of AS (data on file). Further in vitro studies showed that a moderate abrasivity toothpaste containing 1% w/w SS and 5% w/w STP resulted in similar plaque removal and statistically significantly greater stain removal than a higher abrasivity toothpaste containing 16% w/w AS and 5% w/w STP (data on file). It is of interest to explore if these in vitro observations translate into a clinical setting.

The primary objective of this early-phase development study (Phase 1 type) was to evaluate ranking order in extrinsic dental stain removal after brushing twice-daily for 8 weeks with one of either two experimental toothpastes containing SS and or one of either two reference toothpastes containing AS (detailed in Table 1). This study was not designed to demonstrate the specific contribution that SS or STP have on the overall reduction of stain and plaque.

**Table 1:** Treatment groups

Group^a^	Silica content	%STP	RDA	Name
RDA ~ 38, 0.5% SS	0.5% w/w, spherical	0%	~38	Experimental product
RDA ~ 58, 1% SS/5% STP	1% w/w, spherical	5%	~58	Experimental product
RDA ~ 36, 6% AS	6% w/w, abrasive	0%	~36	Sensodyne^®^ Pronamel daily protection—mint essence^b^
RDA ~ 166, 16% AS/5% STP	16% w/w, abrasive	5%	~166	Sensodyne^®^ extra whitening^b^

^a^All study products contained 5% w/w potassium nitrate and 0.2542% w/w sodium fluoride

^b^USA-marketed product, GSK Consumer Healthcare, Brentford, UK

## Materials and methods

This was an 8-week, randomised, examiner-blind, stratified, parallel-group study carried out at a USA-based clinical research facility in compliance with the Declaration of Helsinki. An independent institutional review board approved the protocol (US Investigational Review Board, Miami, FL, USA; U.S.IRB2017SRI/10). The trial was registered at clinicaltrials.gov (NCT03267511).

### Participants

Eligible participants (18–65 years) had ≥20 natural teeth (including 12 anterior teeth with restorative materials covering less than 25% of the tooth) and ≥4 anterior teeth with extrinsic dental stain due to dietary factors (in the investigator’s opinion) and facial surfaces gradable using the MacPherson modification of the Lobene stain index (MLSI).^25^

Exclusion criteria included: pregnancy; breastfeeding; antimicrobial or staining mouthwash use; antibiotic use within 30 days of screening or prior to baseline; a medical condition or medication/product use that could confound study results; hypersensitivity/intolerance to study materials; gross periodontal disease or treatment within 12 months, dental prophylaxis within 8 weeks, or scaling, root planning or bleaching/whitening product use (excluding daily-use whitening toothpastes) within 3 months of screening. Tooth-specific exclusions included: appeared non-vital; caries (or treatment of) within 12 months of screening; exposed dentine or hypo/hyperplastic areas that could impact stain grading; deep, defective or facial restorations; an abutment for fixed/removable partial dentures; full crown/veneer, orthodontic appliances, partial dentures, fixed retainers or cracked enamel; surface irregularities/discolouration or high levels of calculus deposits.

### Clinical procedures and study products

At screening, participants gave written informed consent to study participation. Demography, medical history and current medications were recorded, followed by oral soft (OST) and hard (OHT) tissue examinations. Participants brushed their anterior teeth with a wetted toothbrush (Oral-B^®^ Sensi Soft Manual Toothbrush; Proctor & Gamble, Cincinnati, USA) to remove external debris, then teeth were air dried followed by a MLSI stain assessment of the 12 anterior teeth. Facial surfaces were divided into two regions: ‘gingival’ (~2 mm wide band adjacent to gingiva free margin) and ‘body’ (sub-divided into distal, body and mesial areas). Stain was scored by Area (*A*), from 0 = no stain to 3 = stain covering more than 2/3 of the region and Intensity (*I*), from 0 = no stain to 3 = heavy stain.^25^ Overall MLSI (*A* × *I*) was derived by tooth region then averaged over all sites. Interproximal analysis comprised mesial and distal areas. MLSI assessments were performed by a single clinical examiner, in the same room, with consistent light levels.

Eligible participants used their own toothpaste between screening and baseline (1–14 days) and attended Visit 2 having refrained from oral hygiene procedures for ≥6 h and eating and drinking (except water) for ≥2 h. After OST/OHT examinations, a full MLSI stain assessment was undertaken. Participants with sufficient stain on facial surfaces of anterior teeth were stratified according to low (<15) or high (≥15) baseline MLSI (*A* × *I*) scores on the four teeth with greatest stain, which became the assessment teeth throughout. Participants brushed with a standard fluoride toothpaste (USA-marketed Colgate^®^ Cavity Protection; Colgate-Palmolive Co., New York, NY, USA) before leaving the study site. After Visit 2, participants used only the provided study products and abstained from chewing gum, using any other dental products and using tobacco/nicotine-containing products.

Participants attended Visit 3 a day after Visit 2 having refrained from all oral hygiene procedures for 24 h (+6/−2 h) and from eating and drinking (except water) for at least 2 h. Plaque was disclosed using Trace^®^ disclosing solution (Young Dental Manufacturing, Earth City, MO, USA) then all gradable teeth (excluding third molars) were assessed using the Turesky modification of the Quigley Hein Index (TPI).^26^ Participants with an overall pre-brushing mean TPI of ≥2.0 continued in the study.

Eligible participants were randomised to one of four treatment groups (detailed in Table 1) according to a randomisation block design, generated by an independent statistics agency, with equal allocation to each MLSI stratum. Participants brushed their teeth under supervision using a ribbon of their allocated toothpaste covering the entire head of the provided toothbrush for 1 timed minute. Plaque was re-disclosed prior to a second post-brushing TPI assessment. Toothpaste tubes were overwrapped in white vinyl to conceal labelling. The study examiner, statistician and anyone who could influence study outcomes were blinded to product allocation.

At home, participants brushed morning and evening for 8 weeks, recording use in a provided diary. They returned after 2, 4 and 8 weeks for an OST examination and MLSI assessment and the day after the Week 8 visit to assess 24-h plaque (TPI) before/after supervised brushing.

Five participants were randomly selected for repeat MLSI/TPI assessments across each assessment window. Repeatability was determined in replicate examinations performed by the same dental examiner. A Fleiss–Cohen weighted kappa coefficient (*κ*) was calculated to assess intra-examiner reliability: excellent reliability if *κ* > 0.75 fair to good if 0.4 ≤ *κ* ≤ 0.75; poor if *κ* < 0.4.

Safety was assessed based on OHT/OST examinations and occurrence of adverse events (AEs) from Visit 2 until 5 days following last study product administration.

### Statistical analysis

No formal sample size was proposed as this was an early-phase development study. Sufficient participants were screened to ensure at least 124 could be randomised to treatment and ~120 (30 per treatment arm) would complete the study. Nevertheless, it was calculated that a sample size of 30 participants in each treatment group had 80% power to detect a difference in means of 0.127 in overall MLSI assuming the standard deviation (SD) was 0.172 using a 2-group *t*-test with a 0.050 two-sided significance level, based on a previous study.^20^

The efficacy analysis was performed on a modified intent-to-treat (mITT) population, defined as all randomised participants who received at least one study treatment dose and had at least one post-baseline efficacy measurement. The safety population included all randomised participants who received a study product at least once.

The primary efficacy objective was to evaluate rank order of study products in terms of level of extrinsic dental stain removal after 8 weeks’ twice-daily brushing. This was achieved by comparing adjusted mean change from baseline in overall MLSI (*A* × *I*); calculated by analysis of covariance [ANCOVA] with treatment as fixed effect and baseline overall MLSI [*A* × *I*] as a covariate) and confidence intervals (CIs) for the means along with their change from baseline plots of MLSI over time. Secondary efficacy objectives, analysed as per the primary, were change from baseline in overall MLSI (*A* × *I*) after 8 weeks for RDA ~ 38, 0.5% SS compared with RDA ~ 36, 6% AS and for RDA ~ 58, 1% SS/5% STP compared with RDA ~ 166, 16% AS/5% STP.

Exploratory objectives included rank order of TPI after a single use and after 8 weeks and change from baseline in overall interproximal MLSI (*A* × *I*), overall area and overall intensity after 2, 4 and 8 weeks’ brushing. Change from baseline in TPI variables was analysed using ANCOVA (fixed effects: treatment, MLSI stratification; covariate: baseline pre-treatment TPI). Stain variables were analysed as per the primary efficacy variable using the appropriate baseline values as covariates.

Assumption of normality and homogeneity of variance in the ANCOVA model was considered satisfied. Missing data were not replaced or imputed. Participants who withdrew were included in the statistical analyses up to discontinuation point. Statistical analyses were performed using SAS Studio version 9.4 (SAS Institute Inc, Cary, NC, USA).

## Results

**Fig. 1:**
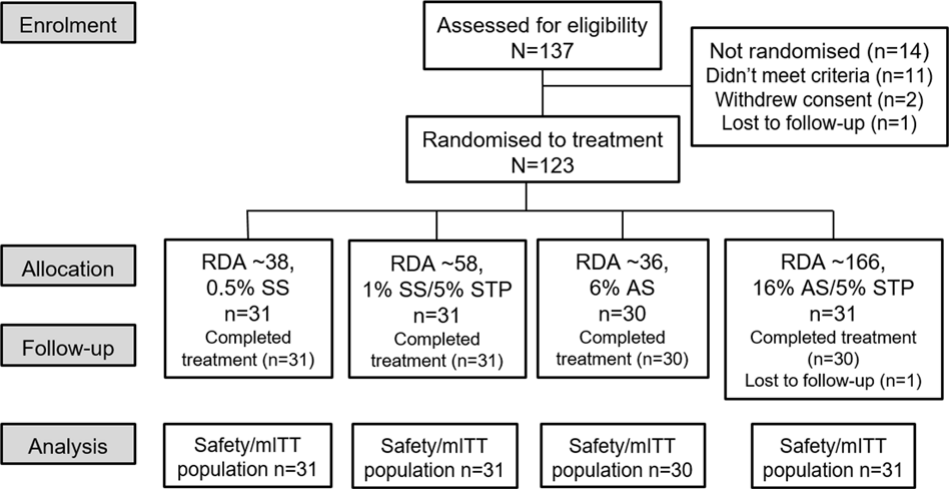
Study flow

The first participant was enroled in September 2017; the last completed the study in December 2017. Of 137 screened participants, 123 were randomised to treatment, received the study product and had at least one post-baseline efficacy measurement (Fig. 1). Overall, mean age was 44 years (range 21–65 years) and more participants were female (74.0%). Treatment groups were well balanced at baseline (Table 2). The MLSI (I), MLSI (A) and TPI scores demonstrated high repeatability (weighted *κ* = 0.98, 0.98 and 0.95, respectively).

**Table 2:** Baseline demographics and characteristics (safety population)

	RDA ~ 38, 0.5% SS (*n* = 31)	RDA ~ 58, 1% SS/5% STP (*n* = 31)	RDA ~ 36, 6% AS (*n* = 30)	RDA ~ 166, 16% AS/5% STP (*n* = 31)
Gender, number (%)				
Female	26 (83.9)	23 (74.2)	19 (63.3)	23 (74.2)
Male	5 (16.1)	8 (25.8)	11 (36.7)	8 (25.8)
Race, number (%)				
African American	6 (19.4)	3 (9.7)	3 (10.0)	2 (6.5)
Asian	1 (3.2)	1 (3.2)	0	1 (3.2)
White	24 (77.4)	27 (87.1)	27 (90.0)	28 (90.3)
Mean age, years (SD)	45.5 (11.32)	46.2 (12.42)	41.3 (11.25)	42.9 (10.57)
Stratification, number (%)				
Overall MLSI (A × I) low (<15)	3 (9.7)	3 (9.7)	2 (6.7)	3 (9.7)
Overall MLSI (A × I) high (≥15)	28 (90.3)	28 (90.3)	28 (93.3)	28 (90.3)

### Efficacy

At baseline, mean overall MLSI (A × I) scores (SD) for each group were: RDA ~ 38, 0.5% SS toothpaste: 1.92 (1.278); RDA ~ 58, 1% SS/5% STP toothpaste: 2.03 (1.153); RDA ~ 36, 6% AS toothpaste: 1.73 (0.735); and RDA ~ 166, 16% AS/5% STP toothpaste: 1.67 (0.837). Raw mean overall MLSI (A × I) scores at Weeks 2, 4 and 8 are shown in Supplementary Table S1.

**Fig. 2:**
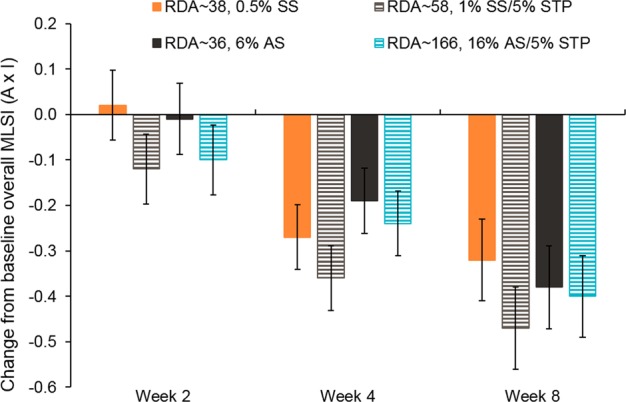
Adjusted mean change from baseline in overall MLSI (*A* × *I*)^a^ (± standard error) at each timepoint (mITT population). ^a^From analysis of covariance model with treatment as fixed effect and baseline overall MLSI score as a covariate. A negative change from baseline in overall MLSI score indicates improved stain control

Adjusted mean changes from baseline in overall MLSI (*A* × *I*) score over time are shown in Fig. 2. All groups decreased from baseline to Week 8 where adjusted mean (standard error [SE]) changes from baseline in overall MLSI (*A* × *I*) scores were: RDA ~ 38, 0.5% SS toothpaste: −0.32 (0.090); RDA ~ 58, 1% SS/5% STP toothpaste: −0.47 (0.090); RDA ~ 36, 6% AS toothpaste: −0.38 (0.091) and RDA ~ 166, 16% AS/5% STP toothpaste: −0.40 (0.090). Based on numerical change in overall MLSI (*A* × *I*) score from baseline to Week 8 (primary endpoint) and Week 4 (exploratory endpoint), ranking order of study products in ability to remove extrinsic dental stain was RDA ~ 58, 1% SS/5% STP toothpaste >RDA ~ 166, 16% AS/5% STP toothpaste >RDA ~ 36, 6% AS toothpaste >RDA ~ 38, 0.5% SS toothpaste.

For the secondary endpoint, the difference in adjusted mean change from baseline at Week 8 in overall MLSI (*A* × *I*) for RDA ~ 38, 0.5% SS toothpaste versus RDA ~ 36, 6% AS toothpaste was 0.06 (95% CI −0.19 to 0.31), non-significantly favouring the latter. The difference between RDA ~ 58, 1% SS/5% STP toothpaste and RDA ~ 166, 16% AS/5% STP toothpaste was −0.07 (95% CI −0.33 to 0.18), non-significantly favouring the former.

Ranking order at Week 8 for exploratory analyses for interproximal MLSI (*A* × *I*), overall *A* and overall *I* were similar to that of the primary and secondary endpoints (data not shown). Ranking order for plaque (overall TPI; Supplementary Table S2) was: RDA ~ 166, 16% AS/5% STP toothpaste >RDA ~ 58, 1% SS/5% STP toothpaste >RDA ~ 38, 0.5% SS toothpaste >RDA ~ 36, 6% AS toothpaste.

### Safety

Study products were generally well tolerated. There were no deaths, incidents, serious AEs or withdrawals due to treatment-emergent AEs (Table 3). All were mild or moderate in intensity and had resolved by study completion.

**Table 3:** TEAEs (safety population)

	RDA ~ 38, 0.5% SS (*n* = 31)		RDA ~ 58, 1% SS/5% STP (*n* = 31)		RDA ~ 36, 6% AS (*n* = 30)		RDA ~ 166, 16% AS/5% STP (*n* = 31)	
	*n* (%)	nAE	*n* (%)	nAE	*n* (%)	nAE	*n* (%)	nAE
At least one TEAE	3 (9.7)	4	1 (3.2)	1	2 (6.7)	2	0	0
At least one oral TEAE	3 (9.7)	4	1 (3.2)	1	2 (6.7)	2	0	0
Oral treatment-related TEAE	1 (3.2)	2	1 (3.2)	1	2 (6.7)	2	0	0
Lip ulcer	1 (3.2)	1	1 (3.2)	1	0	0	0	0
Mouth ulcer	0	0	0	0	1 (3.3)	1	0	0
Palatal ulcer	0	0	0	0	1 (3.3)	1	0	0
Angular cheilitis	1 (3.2)	1	0	0	0	0	0	9

*n* (%) number (percentage) of participants, *nAE* number of adverse event

## Discussion

This early-phase development study examined extrinsic dental stain removal of toothpastes formulated with low levels of SS designed to provide enhanced cleaning properties compared to toothpastes containing standard AS concentrations. Stain was assessed on facial surfaces of four anterior teeth as these surfaces are considered the most aesthetically important with respect to dental stain accumulation. The selection of four teeth allowed teeth and surfaces with a greater propensity for stain accumulation to be included in overall MLSI (*A* × *I*) scores, enabling greater overall differences to be observed if they existed. Use of four anterior teeth was a modification from previous studies where 12 anterior teeth were assessed.^19–22,27^ While this was considered appropriate for an early-phase development study without formal comparisons, assessment of fewer teeth may have contributed to the large SEs observed for mean MLSI scores between treatment groups and variability at baseline (the planned SD was 0.172; the observed SD was ~0.490). This study was only powered to observe trends and lower participant numbers is a further factor likely to have contributed to the observed SDs.

The marketed reference toothpastes had a similar formulation chassis and were chosen to represent toothpastes with low (RDA ~ 36) and high (RDA ~ 166) abrasivity values, respectively containing 6% and 16% w/w standard AS. In contrast, the experimental toothpastes contained only 0.5% (RDA ~ 38) and 1% (RDA ~ 58) w/w of the novel, structured, high-cleaning SS. Despite a far lower concentration of this silica, the ranking order in ability to remove extrinsic dental stain put the 1% SS toothpaste above both marketed toothpastes based on numerical change in overall MLSI (A × I) score by Week 8. In terms of plaque removal efficacy after a single brushing after 8 weeks’ use, while the 16% AS toothpaste showed the greatest change in TPI score, this was closely followed by the 1% SS toothpaste, with the 6% AS toothpaste showing the lowest change in plaque removal score, below that of the 0.5% SS toothpaste. This again indicates the possible utility of low concentrations of SS in a toothpaste to provide comparable cleaning to toothpastes with higher concentrations of AS.

This study was not designed to detect if stain removal capability of the 1% SS and 16% AS toothpastes could be attributed to either the silica, the 5% STP or both working together. While both STP-containing toothpastes ranked above the non-STP toothpastes, they also contained higher SS or standard AS levels, so no clear conclusions can be made regarding the relative contribution of STP to the stain removal scores. It is of considerable scientific interest to further understand this technology and explore which feature of the toothpaste is contributing most to the relative stain removal efficacy. All study products were generally well tolerated.

## Conclusions

Although relatively small reductions of stain removal were observed, a trend was shown in this early-phase development study that a toothpaste with a combination of 1% novel, structured, high-cleaning SS plus 5% STP facilitates higher stain removal than a toothpaste with 16% AS plus 5% STP and non-STP-containing toothpastes with 0.5% SS or 6% AS. It is of interest to explore the relationships between these physical and chemical cleaning agents further and increase understanding of the relative contribution of different levels of SS, with and without STP. Toothpastes with reduced abrasivity are considered to be of benefit for people with dentin hypersensitivity.^16^ Given that promising results were observed using relatively low SS concentrations in comparison to higher AS concentrations, it is of interest to utilise this technology to develop lowered abrasivity toothpastes for people with dentin hypersensitivity.

## Supplementary information

Supplementary Information
