# Supplementary material for: Stain control with two experimental dentin hypersensitivity toothpastes containing spherical silica: a randomised, early-phase development study
Source: BDJ Open. 2019 Jun 6;5:8. doi: 10.1038/s41405-019-0016-x (PMC6554270; doi:10.1038/s41405-019-0016-x)
Supplement: Supplementary file 1 — Supplementary Information [file 41405_2019_16_MOESM1_ESM.pdf]

## SUPPLEMENTARY INFORMATION

**Supplementary Table S1.** Mean overall Macpherson modification of the Lobene stain index (A × I) (± standard deviation) at each timepoint (modified intent-to-treat population)

| | **RDA~38, 0.5% SS (n=31)** | **RDA~58**, **1% SS/5% STP (n=31)** | **RDA~36, 6% AS (n=30)** | **RDA~166**, **16% AS/5% STP (n=31)** |
|---|---|---|---|---|
| Baseline | 1.92 (1.278) | 2.03 (1.153) | 1.73 (0.735) | 1.67 (0.837) |
| Week 2 | 1.91 (1.059) | 1.85 (0.833) | 1.76 (0.710) | 1.63 (0.665) |
| Week 4 | 1.61 (0.922) | 1.59 (0.645) | 1.58 (0.656) | 1.50 (0.635) |
| Week 8 | 1.57 (0.943) | 1.48 (0.782) | 1.40 (0.712) | 1.34 (0.694) |

AS: abrasive silica; RDA: Relative dentin abrasivity; SS: spherical silica; STP: sodium tripolyphosphate

**Supplementary Table S2.** Mean overall Turesky Plaque Index (TPI) score and change from pre-brushing to post-brushing adjusted mean overall TPI score[a] at baseline and Week 8 (modified intent-to-treat population)

| | **RDA~38, 0.5% SS (n=31)** | **RDA~58, 1% SS/5% STP (n=31)** | **RDA~36, 6% AS (n=30)** | **RDA~166, 16% AS/5% STP (n=31)** |
|---|---|---|---|---|
| **Baseline** | | | | |
| Raw mean pre-brushing score (SD) | 3.08 (0.370) | 2.84 (0.275) | 3.01 (0.359) | 2.94 (0.297) |
| Raw mean post-brushing score (SD) | 2.48 (0.414) | 2.15 (0.368) | 2.40 (0.459) | 2.24 (0.336) |
| Adjusted change from pre-brushing to post-brushing score (SE) | -0.61 (0.042) | -0.69 (0.042) | -0.61 (0.042) | -0.69 (0.041) |
| 95% Confidence Intervals | -0.69, -0.52 | -0.77, -0.61 | -0.69, -0.53 | -0.77, -0.61 |
| P-value[a] | <0.0001 | <0.0001 | <0.0001 | <0.0001 |
| **Week 8** | | | | |
| Raw mean pre-brushing score (SD) | 3.22 (0.363) | 2.86 (0.266) | 3.11 (0.358) | 2.85 (0.324) |
| Raw mean post-brushing score (SD) | 2.60 (0.446) | 2.21 (0.429) | 2.61 (0.406) | 2.22 (0.354) |
| Adjusted change from pre-brushing to post-brushing score (SE) | -0.46 (0.059) | -0.66 (0.060) | -0.38 (0.060) | -0.73 (0.059) |
| 95% Confidence Intervals | -0.58, -0.34 | -0.77, -0.54 | -0.50, -0.26 | -0.84, -0.61 |
| P-value[a] | <0.0001 | <0.0001 | <0.0001 | <0.0001 |

[a]From analysis of covariance model with treatment and Macpherson modification of the Lobene stain index stratification as fixed effects and appropriate pre-brush TPI as a covariate. A reduction in TPI score indicates improved plaque control.

AS: abrasive silica; RDA: relative dentin abrasivity; SD: standard deviation; SE: standard error: SS: spherical silica; STP: sodium tripolyphosphate
